# A Security Scheme Based on Intranal-Adding Links for Integrated Industrial Cyber-Physical Systems

**DOI:** 10.3390/s21082794

**Published:** 2021-04-15

**Authors:** Dandan Zhao, Can Liu, Hao Peng, Juan Yu, Jianmin Han

**Affiliations:** College of Mathematics and Computer Science, Zhejiang Normal University, Jinhua 321004, China; ddzhao@zjnu.edu.cn (D.Z.); liucan@zjnu.edu.cn (C.L.); yujuan@zjnu.edu.cn (J.Y.); hanjm@zjnu.cn (J.H.)

**Keywords:** cyber-physical system, adding strategies, cascading failure, robustness

## Abstract

With the advent of the Internet of Everything era, the Industrial Internet is increasingly showing mutual integration and development. Its core framework, the industrial CPS (Cyber-Physical Systems), has received more and more attention and in-depth research in recent years. These complex industrial CPS systems are usually composed of multiple interdependent sub-networks (such as physical networks and control networks, etc.). Minor faults or failure behaviors between sub-networks may cause serious cascading failure effects of the entire system. In this paper, we will propose a security scheme based on intranal-adding links in the face of the integrated and converged industrial CPS system environment. Firstly, by calculating the size of the largest connected component in the entire system, we can compare and analyze industrial CPS systems’ security performance under random attacks. Secondly, we compare and analyze the risk of cascading failure between integrated industrial CPS systems under different intranal-adding link strategies. Finally, the simulation results verify the system security strategy’s effectiveness under different strategies and show a relatively better exchange strategy to enhance the system’s security. In addition, this paper’s research work can help us design how to further optimize the interdependent industrial CPS system’s topology to cope with the integrated and converged industrial CPS system environment.

## 1. Introduction

**Figure 1:**
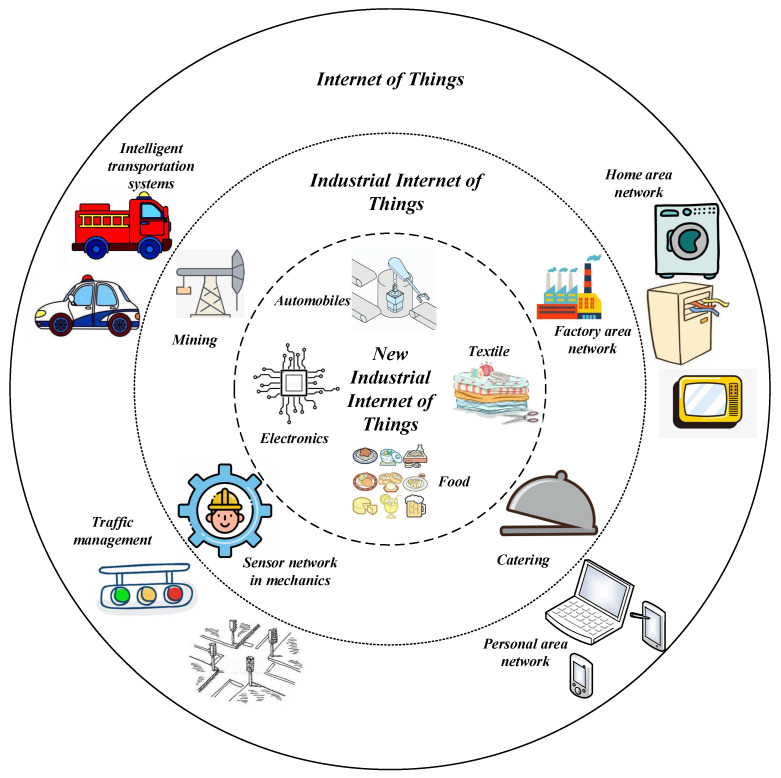
Application scenarios of the industrial internet of things.

Cyber-physical systems (CPS) integrate computing components, networks, and physical processes into specific environments [1,2,3]. Many social systems can be abstracted as CPS. The CPS plays an irrreplaceable role in a broad range. For instance, the power grid system and vehicle network system are considered as the CPS [2,4,5,6,7]. Preserving the robustness of these social systems is essential. One typical CPS can be abstracted as cyber networks and physical networks. These networks are integrated and constructed as an interdependent network. An interdependent network that is built by several networks is more accessible than a single network [8,9,10]. As shown in Figure 1, the industrial internet of things is a part of the internet of things and many standard systems are in the range of the industrial internet of things. The industrial internet of things can also be abstracted as cyber networks integrate physical networks similar to CPSs. Computers are used to control and monitor physical networks. The physical networks can exchange data with other systems [11].

### 1.1. Interdependent Networks

The social networks link nodes from several networks with specific rules. Therefore, the social system’s scale increases from a single network to interdependent networks. There are three interdependent network models which are established in existing studies. All of these models have been widely used in studies.

Buldyrev et al. [12,13] construct a ‘one-to-one correspondence’ model to specify the relationship of some basic systems. In this model, Buldyrev assumes that interdependent networks consist of two complex networks, which are named network *A* and network *B*. Node *i* in the network *A* has only one dependent link from node *j* which is in the network *B* and *j* has one independent link with *i*. This model is the simplest correspondence model.

To better the fidelity of the connection of network models, the ‘one-to-multiple correspondence’ model is built [14,15,16,17]. In this model, node *i* in the network *A* has just one dependent link from node *j*, which belongs to the network *B*. However, *j* has several independent links from $A^{\prime }$ nodes. This correspondence model is more complex than the ‘one-to-one correspondence’ model. Some real-world networks obey this correspondence model.

The especially complex correspondence model is ’multiple-to-multiple correspondence’ [18,19]. Scholars observe that node *i* in the network *A* has at least two independent links from *B*. However, node *j* has more than one independent link from *A*. Thus, this model is greater in scale and complicated.

### 1.2. Cascading Failure

Scholars discuss detailed cascading failure’s processes in [12,20]. In [12], they design an interdependent network by two single networks and assume random attacks starting the failure. They consider the necessary and sufficient conditions for normal working nodes. In the following simulation, we follow these conclusions from [12] that a normal working node must satisfy these two conditions at the same time:

(i) This node has more than one dependent link from normal working nodes;
(ii) This node belongs to the giant component.

Because some nodes do not follow the above two conditions, these nodes and within links are removed. The cascading failure recursive propagation is in different networks. When the failure stops, the statement of the system will be one of two conditions:

(i) One is that the system is collapsing;
(ii) The other statement is that there are still some nodes working normally. The entire system will be in a stable state.

In the study of resisting cascading failure, five approaches to enhance the robustness of the homogeneous interdependent network are applied. They protect crucial network nodes from strengthening the reliability of networks [21,22]. Making nodes’ autonomy enhance robustness is costly [18,23]. Adjusting dependency link allocation and refiguring the topology of the network by rewiring [7,24,25,26,27] are also applied in networks. However, these two methods are only suitable for designing networks. Adding links in systems is simulated in [28,29,30]. They find that adding links can improve a CPS’s reliability. The above methods are performing more significant effects in improving the reliability of an interdependent model. Nevertheless, they have some limitations on practical applications.

In Section 2, we specify the model of the CPS, which is built in Section 4. In Section 3, we describe seven intra-links adding strategies and describe the adding links processes of these strategies in the CPS. Section 4 is the simulation figures description and results. Section 5 is conclusions and works which we are exploring in the future.

## 2. Mathematical Model

**Figure 2:**
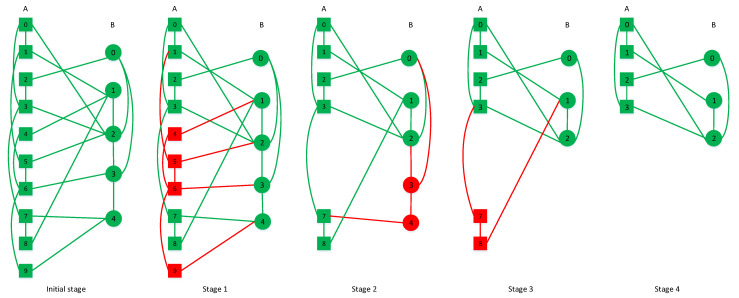
The processes of cascading failures in an interdependent network. Ten nodes and five nodes are in networks *A* and *B*. Node $A_{5}$ in the network *A* triggers the cascading failure of the entire system. At stage 1, all links with node $A_{5}$ are failed and removed. Thus, node $A_{4}$, $A_{6}$, and $A_{9}$ are disconnected from the giant component in the network *A*. At the second stage, node $A_{4}$, $A_{6}$, and $A_{9}$ have removed all links. Node $B_{3}$ loses supporting links from network *A* and $B_{4}$ is excluded from the giant component. Thus, these two nodes fail. All nodes $B_{3}$, $B_{4}$ and their links are deleted in stage 3. Then, the network *B* splits. Node $A_{7}$ fails as it loses its dependent nodes. Node $A_{8}$ fails since it is part of the giant component. In the final stage, the cascading failure stops propagating on this model. Only several nodes can operate properly.

At first, we describe CPS models in detail, which are simulated in Section 4. Next, the node number’s formulas during cascading failure processes are shown [12]. In the end, we show a simple cascading failure model within the ‘one-to-multiple correspondence’ model in Figure 2.

In [31], scholars propose a classification for CPSs. Besides characteristic behaviors of the CPS, different algorithms to model the intra-connection and inter-connection between networks are researched in [32,33].

### 2.1. Interdependent Model

In our simulation, we construct a CPS model composed of two complex networks: the network *A* and network *B*. Intra-links order complex network’s degree distribution. All inter-links are randomly connecting nodes with different networks [14,20]. All links are non-directional to these CPS models. This setting means that, if node *i* has an inter-link with node *j*, then these two nodes depend on each other.

Both the Erdös-Rényi (ER) network and scale-free (SF) network are systematically studied [12,34]. In interdependent network models, network *A* and network *B* are ER networks or SF networks. If the network is an ER network, the network nodes’ degree must order binomial distribution. The SF network degree distribution is following the power-law distribution. The formula of power-law degree distribution is $P\left(k\right)\propto k^{-\gamma }$. In this formula, P(k) is the degree distribution and γ is the power-law exponent.

As explained in Section 1, the ‘one-to-multiple’ model is preferred to model independent relationships between power stations and control equipment. To properly simulate the power grid system, the ‘one-to-multiple correspondence’ is a better choice for modeling inter-links’ connection relationships. The inter-links’ coupled ratio is set at 3:1. It denotes that node *i*, which belongs to the network *A*, relies on node *j* of network *B*, but *j* includes three independent nodes from *A*. If *j* fails, *i* will fail. However, if *i* fails, *j* may not fail. The condition for *j* fails is that all independent links with it fail.

### 2.2. Mathematical Formulation

With the introduction of the cascading failure setting in Section 1, scholars derive the formulas of the number of nodes in all cascading failure processes. The notations of this section are shown in Table 1. For a ‘one-to-one correspondence’ model, nodes’ number at a stable state in the network *A* and network *B* is:

(1) $$\left\{\begin{array}{cc} x & =g_{A}\left(y\right)p\\ y & =g_{B}\left(x\right)p \end{array}\right.$$

In the following simulation models, existing studies of cascading failure follow. The cascading failure is triggered by a small fraction of the nodes’ failure. Thus, the most popular assumption is that the random attack occurs in the network *A* and the number of $\left(1-p\right)N_{A}$ nodes failed—following this assumption, by removing $\left(1-p\right)N_{A}$ nodes at random from the network *A* as random attacks. At the same time, all links within failed nodes are deleted. The remaining nodes’ number of network *A* are:

(2) $$N_{A1}^{\prime }=p\cdot N_{A}=\mu^{\prime }\cdot N_{A}$$

The fraction of nodes in the giant component in $N_{A1}^{\prime }$ is:

(3) $$N_{A1}=g_{A}\left(\mu_{1}^{\prime }\right)\cdot N_{A1}^{\prime }=\mu_{1}^{\prime }\cdot g_{A}\left(\mu_{1}^{\prime }\right)\cdot N_{A1}=\mu_{1}\cdot N_{A}$$

Each node in the network *B* relies on three nodes from the network *A*. As in the above settings, one node in network *B* will fail if it does not have inter-links in the second stage. The normal working nodes in network *B* are [20]:

(4) $$\begin{array}{cc} \; & N_{B2}^{\prime }=\left[1-{\left(1-\mu_{1}\right)}^{3}\right]N_{B}=\mu_{2}^{\prime }\cdot N_{B} \end{array}$$

(5) $$\begin{array}{cc} \; & \mu_{2}^{\prime }=1-{\left(1-\mu_{1}\right)}^{3}=\mu_{1}^{3}-3\mu_{1}^{2}+3\mu_{1}=\left(\mu_{1}^{2}-3\mu_{1}+3\right)\mu_{1}^{\prime }g_{A}\left(\mu_{1}^{\prime }\right) \end{array}$$

As failed nodes and their intra-links are erased from the system, the network *B* separates into several components. Nodes in the giant component will be preserved while the others are deleted. The fraction of preserved network *B* nodes is:

(6) $$\begin{array}{cc} \; & N_{B2}=g_{B}\left(\mu_{2}^{\prime }\right)\cdot N_{B2}^{\prime }=\mu_{2}^{\prime }\cdot g_{B}\left(\mu_{2}^{\prime }\right)\cdot N_{B}=\mu_{2}\cdot N_{B} \end{array}$$

(7) $$\begin{array}{cc} \; & \mu_{2}=\mu_{2}^{\prime }\cdot g_{B}\left(\mu_{2}^{\prime }\right) \end{array}$$

These failed nodes in the network *B* will lead to the cascading failure to network *A*. This failure propagates between the CPS until it stops. The nodes’ number must be the formulas in the steady stage:

(8) $$\left\{\begin{array}{c} \mu_{2i}^{\prime }=\mu_{2i-2}^{\prime }=\mu_{2i+2}^{\prime }\\ \mu_{2i+1}^{\prime }=\mu_{2i-1}^{\prime }=\mu_{2i+3}^{\prime } \end{array}\right.$$

The next stage of the cascading failure is shown in Table 2. With these formulas, the fraction of working nodes at the steady state in the network *A* and *B* is [20]:

(9) $$\begin{array}{cc} \; & \mu_{A_{\infty }}=xg_{A}\left(x\right) \end{array}$$

(10) $$\begin{array}{cc} \; & \mu_{B_{\infty }}=yg_{B}\left(y\right) \end{array}$$

where

(11) $$\left\{\begin{array}{c} x=pg_{B}\left(y\right)\\ y=p\left[{\left(xg_{A}\left(x\right)\right)}^{2}-3xg_{A}\left(x\right)+3\right]g_{A}\left(x\right) \end{array}\right.$$

Equation (11) changed into:

(12) $$x=p\cdot g_{B}\left\{p\left[{\left(x\cdot g_{A}\left(x\right)\right)}^{2}-3xg_{A}\left(x\right)+3\right]\cdot g_{A}\left(x\right)\right\}$$

To perform the cascading failure in a ‘one-to-multiple correspondence’ model, some nodes’ connection of the simulation model is illustrated in Figure 2. The relationship between intra-links and inter-links is illustrated in the figure’s initial stage. When the cascading failure ends, the connection of the system is depicted in stage 4.

## 3. Methodology

In [28], different adding strategies are proposed to enhance the ‘one-to-one correspondence’ model’s reliability. The calculation formulas and implementation method of seven adding strategies applied in the following simulation are given in this section.

NONE implies that nodes do not add intra-links to CPS models. The model’s construction has not been modified.

I. Random adding strategy (RA)

RA is randomly selecting two nodes from the network *A* or network *B*. Then, analyze the connection of intra-links within these two nodes and set one intra-link to link these two nodes. Based on the basic requirements of undirected networks, parallel links and self-loops are forbidden. After one adding operation, there will be one intra-link between two randomly selected nodes. In the entire CPS model, inter-links are not altered after RA strategy. In the simulation, RA is used as a control experiment to contrast with other strategies.

II. Low degree adding strategy (LD)

Degree centrality is widely utilized to calculate the importance of nodes [12,28,35]. It is well-known that the intra-link number of one node is the node’s degree in an undirected network [9,34].

To complete the LD one time, getting all nodes’ degrees first. Then, check the connection relationship of the two minimum degree value nodes of a single network. Finally, add one intra-link between these two selected nodes. In the complete process of adding intra-links, parallel links and self-loops are forbidden.

III. High degree adding strategy (HD)

HD is getting nodes’ degrees first. Then, check the connection relationship of the two highest degree value nodes in one single network. Finally, insert one intra-link between two selected nodes. In the whole process of adding intra-links, parallel links and self-loops are forbidden.

IV. Low betweenness adding strategy (LB)

Nodes’ intra-links constitute several paths in a single network. Betweenness centrality measures nodes’ importance by these paths [28,36]. One node’s betweenness centrality is:

(13) $$B\left(v\right)=\sum_{i\neq j}\frac{\sigma_{ij}\left(v\right)}{\sigma_{ij}}$$

where $\sigma_{ij}$ is the shortest paths number from node *i* to node *j*. $\sigma_{ij}\left(v\right)$ is the shortest paths number from node *i* to node *j* which are through the node *v* [28,34,35]. If one node has a large number of shortest paths to other nodes, this node must be a critical node in the network.

Using the low betweenness adding strategy, getting all nodes’ betweenness centrality values is the first step. Next, evaluate the connection relationship of the two lowest betweenness value nodes of one single network. Adding one intra-link of these two selected nodes is the last step. After the above processes, one time LB is completed. In the complete process of adding intra-links, parallel links and self-loops are forbidden.

V. High betweenness adding strategy (HB)

HB is getting all nodes’ betweenness values first. Then, check the connection relationship of the two highest betweenness value nodes of a single network. Finally, insert one intra-link between these two selected nodes. In the whole process of adding intra-links, parallel links and self-loops are forbidden.

VI. Low eigenvector centrality adding strategy (LEC)

Considering the node’s neighbors to judge the node’s importance is the eigenvector centrality. This centrality has a broad range of applications in daily life, such as satellite cities around economically developed cities and satellite cities around cities with developed tourism. Scholars construct a matrix *A* to indicate the nodes’ intra-link relationship to express one node’s neighbors.. In the matrix *A*, an element $A_{ij}$ denotes whether there is an intra-link between node *i* and node *j*. If $A_{ij}=1$, node *i* and node *j* have one intra-link linking each other; if $A_{ij}=0$, *i* and *j* do not have a path. Because the eigenvector centrality will be changed due to nodes’ neighborhoods, the initial value of node’s eigenvector centrality $x_{i}=1$. The value of $x_{i}$ changes into $x_{i}^{\prime }$ [34,35]:

(14) $$x_{i}^{\prime }=\kappa_{1}^{-1}\sum_{j}A_{ij}x_{j}$$

where $\kappa_{1}$ is the largest eigenvector value in the matrix *A*. If a node is connected to multiple important nodes, the importance of the node will increase.

To finish one time LEC, getting all nodes’ eigenvector centrality value is necessary. Then, check the connection relationship of the two smallest eigenvector value nodes of the single network. Finally, add one intra-link between these two selected nodes. In the entire process of adding intra-links, parallel links and self-loops are forbidden.

VII. High eigenvector centrality adding strategy (HEC)

HEC is getting nodes’ eigenvector centrality values first and checking the connection relationship of the two highest eigenvector centrality value nodes of the single network next. Finally, add one intra-link between these two selected nodes. In the whole process of adding intra-links, parallel links and self-loops are forbidden.

## 4. Results and Discussion

The first subsection explains the parameters of simulation models. In the next, detailed simulation processes are performed. The results are shown at the end of this section.

### 4.1. Parameters

In the following simulation, the CPS models are within the ‘one-to-multiple correspondence’ model. Without loss of generality, the ratio of inter-link relationships is set at 3:1 (the ratio is the largest setting which we can simulate). Based on the above setting, the nodes’ number is $N_{A}=9000$ and $N_{B}=3000$ in two networks. Following previous research settings, both the ER and SF network’s average degree is 〈k〉=4 and γ in the SF network is 3. The real-world network’s degree is closed to 〈k〉=4 [12,34]. If one social network follows the power-law distribution, γ is usually between 2 and 3. When γ=3, this network corresponds to the typical value of the BA model [34]. Both the intra-links and inter-links are non-directional in all models.

In [28], they have found that adding intra-links in double networks yields better performance than in a single network. Thus, we add intra-links in double networks of the CPS model. At first, it is essential to determine the number of added intra-links. $f_{L}$ indicates that the fraction of adding links is:

(15) $$f_{L}=\frac{L^{\prime }}{L_{A}+L_{B}}$$

where $L_{A}$ and $L_{B}$ represent the intra-link’s number in the initial network *A* and *B*. $L^{\prime }$ means adding links’ number. As the nodes number of two networks and the average degree have been determined, the number of intra-links in these networks is 36,000 and 12,000. Therefore, the adding intra-link’s number in the network *A* is $f_{L}\cdot$ 36,000 and in the network *B* is $f_{L}\cdot$ 12,000. We cannot add links indefinitely due to the cost of the system building. As the research of community interaction [37], the most popular node in one system has more than six links with other nodes. In this way, we set 8 as the highest degree of one node in this paper. Therefore, $f_{L}$ cannot be larger than 50%. To clarify the meanings of these parameters, we display these parameters in Table 3.

### 4.2. Reliability Metrics

Apply the random attack into our models to simulate the real-world networks’ attack. To get to know the reliability of one CPS, the metric *G* which means the normal working nodes number fraction after the cascading failure stops is applied:

(16) $$G=\frac{N_{A}^{\prime }+N_{B}^{\prime }}{N_{A}+N_{B}}$$

where $N_{A}^{\prime }$ ($N_{B}^{\prime }$) means the working node’s number in a stable state of the network *A* (*B*). $p_{c}$ is used to reflect the ability of one CPS to fight against random attacks. To clear the meanings of these metrics, we have shown them in Table 3.

### 4.3. Simulation Setup

We write C++ programs to model the cascading process in an interdependent network. We increase 1−p by 0.25 each time to simulate and obtain *G* values. For each value of 1−p, we execute 20 times and get an average of *G* value as the final result to decrease the error. The processes of these simulations are:

- in the first, we build two complex networks (which are named *A* and *B*) to represent an interdependent network. These two networks are selected from the ER and SF network which are generated by binomial distribution and power-law distribution, respectively.
- then, we couple these two networks within the ‘one-to-multiple correspondence’ model. The relationships of inter-links are random connections and the ratio maintains 3:1.
- we apply one adding strategy to one specific model. The relationships of intra-links will change.
- in the further, $\left(1-p\right)N_{A}$ nodes are chosen at random as failed nodes representing the attacked nodes of the network *A*.
- cascading failure propagates between network *A* and *B*. We simulate each propagate’s stage and record the working node’s number of the system at each step.
- finally, we calculate the steady stage node’s number of this entire interdependent network.

### 4.4. Network Size and $p_{c}$

**Figure 3:**
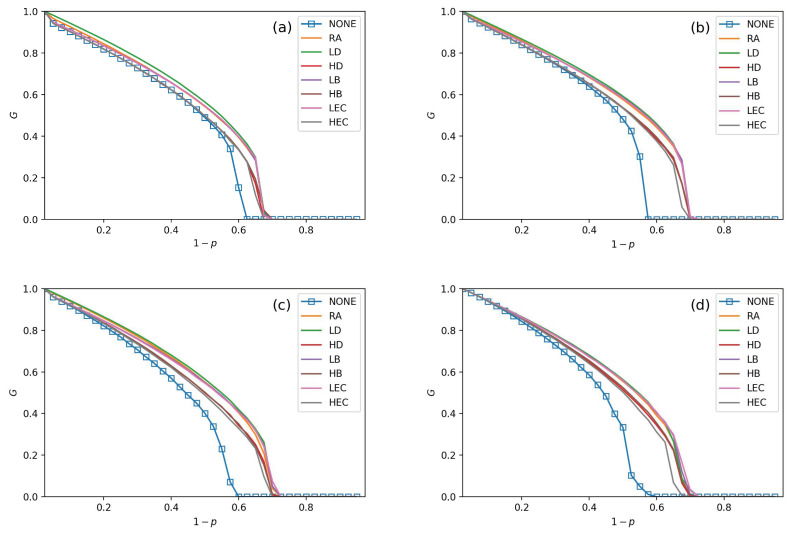
The fraction of function nodes when $f_{L}=15\%$ in ER-ER, ER-SF, SF-ER, and SF-SF system, which is shown in (**a**–**d**).

**Figure 4:**
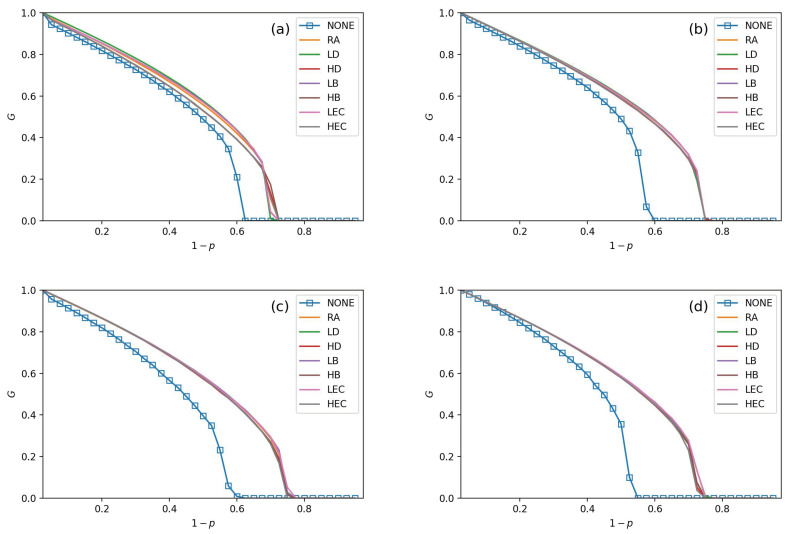
The fraction of function nodes when $f_{L}=25\%$ in ER-ER, ER-SF, SF-ER, and SF-SF system, which is shown in (**a**–**d**).

**Figure 5:**
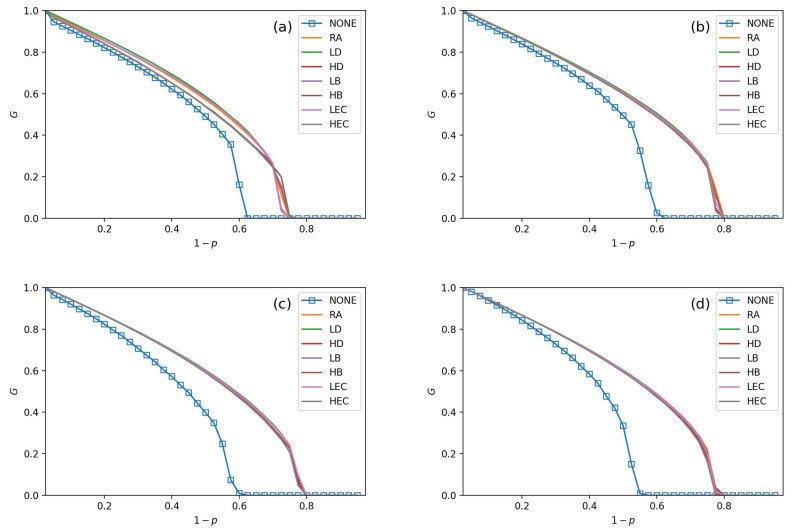
The fraction of function nodes when $f_{L}=35\%$ in ER-ER, ER-SF, SF-ER, and SF-SF system, which is shown in (**a**–**d**).

**Figure 6:**
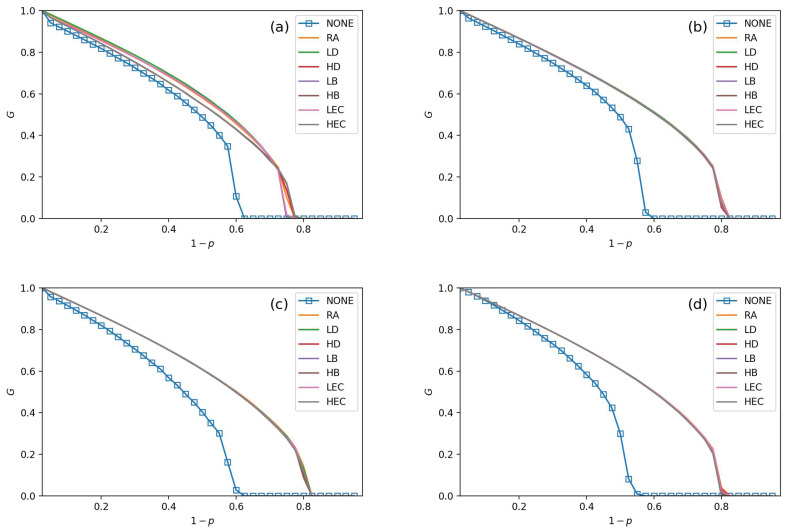
The fraction of function nodes when $f_{L}=45\%$ in ER-ER, ER-SF, SF-ER, and SF-SF system, which is shown in (**a**–**d**).

**Figure 7:**
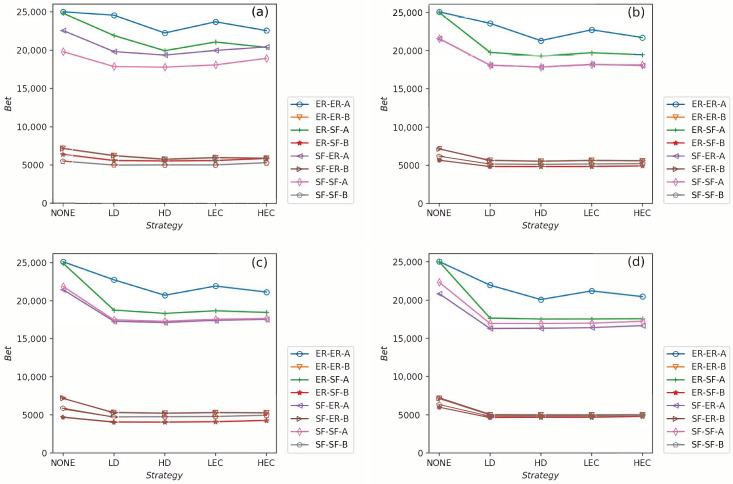
The values of betweenness centrality values in systems when $f_{L}=15\%$, $f_{L}=25\%$, $f_{L}=35\%$ and $f_{L}=45\%$ are shown in (**a**–**d**), respectively.

**Figure 8:**
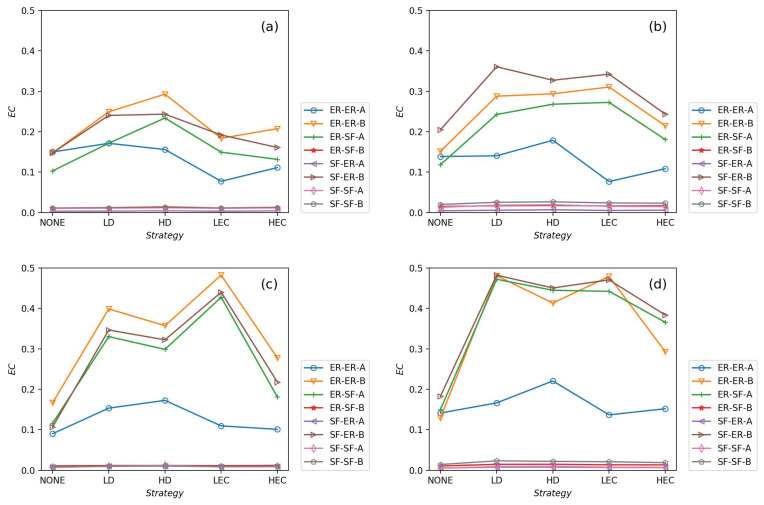
The values of eigenvector centrality values in systems when $f_{L}=15\%$, $f_{L}=25\%$, $f_{L}=35\%$ and $f_{L}=45\%$ are shown in (**a**–**d**), respectively.

Seven adding strategies are applied in models to verify the performance of the CPS. In Figure 3, Figure 4, Figure 5 and Figure 6, the values of $f_{L}$ are setting as 15%, 25%, 35%, and 45%, respectively. In each subfigure, we plot relationships of *G*, $p_{c}$ and 1−p. Then, we plot not adding links (NONE) as a contrast simulation for the other strategies which are detailed in Section 3. From the Figure 3, Figure 4, Figure 5 and Figure 6, we get the following conclusions:

- All strategies make greater robustness of the CPS model. As the value of the $f_{L}$ increases, one CPS model gets more reliable. All seven adding strategies have higher values of *G* and $p_{c}$ than NONE. It means that increasing the number of intra-links can enhance an interdependent network’s reliability. When the number of intra-links increases, the instructions of one network get more complex. The *G* and $p_{c}$ values are increasing when $f_{L}$ increases. The $p_{c}$ nears 0.7 under LEC in Figure 3d. When $f_{L}$ gets 45%, the value of $p_{c}$ is more than 0.8 (in Figure 6d). This conclusion obeys the previous conclusions which are mentioned in [28].
- Under the identical model settings, adding links gets better results in increasing *G* values with low centrality values than by high centrality values, especially when $f_{L}$ is small (in Figure 3). This finding and reason have been shown in [9].
- The LD strategy gets the highest values of *G* and $p_{c}$ in the ER-ER CPS model (shown in all subfigure (a) from Figure 3, Figure 4, Figure 5 and Figure 6). The best choice to enhance *G* and $p_{c}$ is the LEC strategy in the other models. All strategies achieve a similar influence in both *G* and $p_{c}$ values from Figure 4b–d, Figure 5b–d and Figure 6b–d. Adding links by high centrality coincides with low centrality values while $f_{L}>25\%$. It means that the different system’s structures are getting more similar when the value of $f_{L}$ increases.
  The intra-link’s numbers can calculate node degree. To have a more precise understanding of the network structures, we plot average betweenness centrality and average eigenvector centrality value figures after LD, HD, LEC and HEC adding strategy with different $f_{L}$ values in Figure 7 and Figure 8.
- In Figure 7, we graph the relationship of betweenness values and different strategies. We use the form X-Y-Z to illustrate the interdependent networks after adding intra-links [28]. X and Y denote the types of the network of interdependent networks. Z represents the single network of the CPS. We find that the betweenness values of strategies are lower than the original networks (NONE). Gaps between betweennesses among ER-SF, SF-ER, and SF-SF interdependent networks are getting smaller when $f_{L}$ increases. The betweenness values of the *A* network in the ER-ER model are highest under all $f_{L}$.
  In Figure 8, we plot the eigenvector centrality values of different strategies. Eigenvector centrality values of LEC are lower than the other strategies when $f_{L}=15\%$. When $f_{L}=25\%$, $f_{L}=35\%$ and $f_{L}=45\%$, HD and HEC display a tendency to decay compared to their adjacent values. All strategies’ eigenvector centrality values are more closed with $f_{L}$ getting bigger. The eigenvector centrality values of the *A* network are smaller than its correspondence *B* network. The ER network has higher eigenvector centrality values. In Figure 8, the eigenvector centrality values of all strategies are not lower than NONE.
  According to Figure 7 and Figure 8, we can obtain that decreasing the network’s betweenness value and increasing the network’s eigenvector centrality value could enhance network reliability. Nevertheless, a too high or too low cost cannot get the largest *G* and $p_{c}$. Betweenness values and eigenvector centrality values of different interdependent networks are not the same, even if their topologies are similar.

## 5. Conclusions and Future Works

In this paper, increasing the number of intra-links by several strategies to enhance the robustness of different CPSs’ models is our goal. To enrich simulation models, we simulate four kinds of heterogeneous interdependent networks. Then, we add intra-links in interdependent models with different adding links’ ratios. Finally, we record *G* and $p_{c}$ when cascading failure stops. Adding intra-links strategies can enhance the network’s reliability and low centrality values adding methods get better system reliability. Our findings believe that a low degree strategy has the best performance in increasing *G* in the ER-ER system. A low eigenvector strategy is the first choice of scenarios included in SF networks.

Our simulation has some limitations: we should give more theoretical studies about interdependent network’s reliability and different evaluation metrics should be suggested as reflecting the reliability of models. These limitations are directions which we are working towards.

## Figures and Tables

**Table 1:** Notations of Cascading Failures’ Functions.

Symbol	Meaning
*p*	The fraction of nodes which is not failed after initial attacks
$N_{Ai}$, $N_{Bi}$	The fraction of normal nodes of network *A*, *B* in stage *i*
$N_{Ai}^{\prime }$, $N_{Bi}^{\prime }$	The nodes’ number in network *A*, *B* in stage *i*
$\mu_{i}$	The fraction of $N_{Ai}^{\prime }$ ($N_{Bi}^{\prime }$) and $N_{A}$($N_{B}$)
$\mu_{i}^{\prime }$	The fraction of $N_{Ai}$ ($N_{Bi}$) and $N_{A}$($N_{B}$)
$g_{A}$, $g_{B}$	The generating functions of network *A*, *B*

**Table 2:** The Fraction of Working Nodes of the Network *A* and *B*.

	Network *A*	Network *B*
Stage 1	$\mu_{1}^{\prime }=p$ $\mu_{1}=\mu_{1}^{\prime }g_{A}\left(\mu_{1}^{\prime }\right)$	
Stage 2		$\mu_{2}^{\prime }=\left(\mu_{1}^{2}-3\mu_{1}+3\right)\mu_{1}^{\prime }g_{A}\left(\mu_{1}^{\prime }\right)$ $\mu_{2}=\mu_{2}^{\prime }g_{B}\left(\mu_{2}^{\prime }\right)$
Stage 3	$\mu_{3}^{\prime }=\mu_{1}^{\prime }g_{B}\left(\mu_{2}^{\prime }\right)$ $\mu_{3}=\mu_{3}^{\prime }g_{A}\left(\mu_{3}^{\prime }\right)$	
Stage 4		$\mu_{4}^{\prime }=\mu_{1}^{\prime }\left(\mu_{3}^{2}-3\mu_{3}+3\right)g_{A}\left(\mu_{3}^{\prime }\right)$ $\mu_{4}=\mu_{4}^{\prime }g_{B}\left(\mu_{4}^{\prime }\right)$
…	…	…
Stage 2*i*	$\mu_{2i}^{\prime }=\mu_{1}^{\prime }\left(\mu_{2i-1}^{2}-3\mu_{2i-1}+3\right)g_{A}\left(\mu_{2i-1}^{\prime }\right)$ $\mu_{2i}=\mu_{2i}^{\prime }g_{A}\left(\mu_{2i}^{\prime }\right)$	
Stage 2i+1		$\mu_{2i+1}^{\prime }=\mu_{1}^{\prime }g_{B}\left(\mu_{2i}^{\prime }\right)$ $\mu_{2i+1}=\mu_{2i+1}^{\prime }g_{B}\left(\mu_{2i+1}^{\prime }\right)$

**Table 3:** Notations of parameters and metrics.

Symbol	Meaning
$N_{A},N_{B}$	The nodes’ number in network *A*, *B*
〈k〉	The average degree of the network
γ	The parameter of the SF network
$f_{L}$	The fraction of adding intra-links
$L_{A},L_{B}$	The intra-links number of network *A*, *B*
*G*	The functions of normal working nodes after cascading failures
$p_{c}$	The ability of the network to fight random attacks

## Data Availability

Not applicable.

## References

[1] Arafsha F., Laamarti F., El Saddik A. Development of a wireless CPS for gait parameters measurement and analysis. Proceedings of the 2018 IEEE International Instrumentation and Measurement Technology Conference (I2MTC).

[2] Xuhong L., Muhai L. Application of CPS in the Complex Network. Proceedings of the 2011 Fourth International Conference on Intelligent Computation Technology and Automation.

[3] Rana M., Shafiq A., Altaf I., Alazab M., Mahmood K., Chaudhry S.A., Zikria Y.B. (2021). A secure and lightweight authentication scheme for next generation IoT infrastructure. Comput. Commun..

[4] Wang W., Xia F., Nie H., Chen Z., Gong Z., Kong X., Wei W. (2020). Vehicle trajectory clustering based on dynamic representation learning of internet of vehicles. IEEE Trans. Intell. Transp. Syst..

[5] Wang T., Liang Y., Yang Y., Xu G., Peng H., Liu A., Jia W. (2020). An intelligent edge-computing-based method to counter coupling problems in cyber-physical systems. IEEE Netw..

[6] Wang W., Zhao X., Gong Z., Chen Z., Zhang N., Wei W. (2020). An attention-based deep learning framework for trip destination prediction of sharing bike. IEEE Trans. Intell. Transp. Syst..

[7] Zhang J., Yeh E., Modiano E. (2018). Robustness of interdependent random geometric networks. IEEE Trans. Netw. Sci. Eng..

[8] Huang Z., Wang C., Nayak A., Stojmenovic I. (2014). Small cluster in cyber physical systems: Network topology, interdependence and cascading failures. IEEE Trans. Parallel Distrib. Syst..

[9] Peng H., Liu C., Zhao D., Han J. (2019). Reliability analysis of CPS systems under different edge repairing strategies. Phys. A Stat. Mech. Appl..

[10] Wang W., Kumar N., Chen J., Gong Z., Kong X., Wei W., Gao H. (2020). Realizing the Potential of Internet of Things for Smart Tourism with 5G and AI. IEEE Netw..

[11] Peng H., Liu C., Zhao D., Hu Z., Han J., Lu J. (2020). Reliability Analysis of Heterogeneous CPS under Different Swapping Inter-links Strategies. International Symposium on Security and Privacy in Social Networks and Big Data.

[12] Buldyrev S.V., Parshani R., Paul G., Stanley H.E., Havlin S. (2010). Catastrophic cascade of failures in interdependent networks. Nature.

[13] Wu P., Ling Z., Liu L., Jiang Y., Wu H., Dai L. End-to-End Emotional Speech Synthesis Using Style Tokens and Semi-Supervised Training. Proceedings of the 2019 Asia-Pacific Signal and Information Processing Association Annual Summit and Conference (APSIPA ASC).

[14] Huang Z., Wang C., Stojmenovic M., Nayak A. (2014). Characterization of cascading failures in interdependent cyber-physical systems. IEEE Trans. Comput..

[15] Dong G., Chen Y., Wang F., Du R., Tian L., Stanley H.E. (2019). Robustness on interdependent networks with a multiple-to-multiple dependent relationship. Chaos Interdiscip. J. Nonlinear Sci..

[16] Chen L., Yue D., Dou C., Cheng Z., Chen J. (2020). Robustness of cyber-physical power systems in cascading failure: Survival of interdependent clusters. Int. J. Electr. Power Energy Syst..

[17] Chen L., Yue D., Dou C. (2019). Optimization on vulnerability analysis and redundancy protection in interdependent networks. Phys. A Stat. Mech. Appl..

[18] Shao J., Buldyrev S.V., Havlin S., Stanley H.E. (2011). Cascade of failures in coupled network systems with multiple support-dependence relations. Phys. Rev. E.

[19] Jiang J., Xia Y., Xu S., Shen H.L., Wu J. (2020). An asymmetric interdependent networks model for cyber-physical systems. Chaos Interdiscip. J. Nonlinear Sci..

[20] Peng H., Kan Z., Zhao D., Han J. (2019). Security assessment for interdependent heterogeneous cyber physical systems. Mob. Netw. Appl..

[21] Ruj S., Pal A. Analyzing cascading failures in smart grids under random and targeted attacks. Proceedings of the 2014 IEEE 28th International Conference on Advanced Information Networking and Applications.

[22] Nguyen D.T., Shen Y., Thai M.T. (2013). Detecting critical nodes in interdependent power networks for vulnerability assessment. IEEE Trans. Smart Grid.

[23] Cui P., Zhu P., Wang K., Xun P., Xia Z. (2018). Enhancing robustness of interdependent network by adding connectivity and dependence links. Phys. A Stat. Mech. Appl..

[24] Parshani R., Rozenblat C., Ietri D., Ducruet C., Havlin S. (2011). Inter-similarity between coupled networks. Europhys. Lett..

[25] Chattopadhyay S., Dai H., Hosseinalipour S. (2017). Designing optimal interlink patterns to maximize robustness of interdependent networks against cascading failures. IEEE Trans. Commun..

[26] Zhou D., Stanley H.E., D’Agostino G., Scala A. (2012). Assortativity decreases the robustness of interdependent networks. Phys. Rev. E.

[27] Kamran K., Zhang J., Yeh E., Modiano E. Robustness of interdependent geometric networks under inhomogeneous failures. Proceedings of the 2018 16th International Symposium on Modeling and Optimization in Mobile, Ad Hoc, and Wireless Networks (WiOpt).

[28] Ji X., Wang B., Liu D., Chen G., Tang F., Wei D., Tu L. (2016). Improving interdependent networks robustness by adding connectivity links. Phys. A Stat. Mech. Appl..

[29] Jiang Z., Liang M., Guo D. (2011). Enhancing network performance by edge addition. Int. J. Mod. Phys. C.

[30] Beygelzimer A., Grinstein G., Linsker R., Rish I. (2005). Improving network robustness by edge modification. Phys. A Stat. Mech. Appl..

[31] Zhang F., Shi Z., Mukhopadhyay S. (2013). Robustness analysis for battery-supported cyber-physical systems. ACM Trans. Embed. Comput. Syst..

[32] Wang Z., Scaglione A., Thomas R.J. (2010). Generating statistically correct random topologies for testing smart grid communication and control networks. IEEE Trans. Smart Grid.

[33] Derler P., Lee E.A., Vincentelli A.S. (2011). Modeling cyber–physical systems. Proc. IEEE.

[34] Newman M. (2018). Networks.

[35] Peng H., Liu C., Zhao D., Ye H., Fang Z., Wang W. (2020). Security Analysis of CPS Systems Under Different Swapping Strategies in IoT Environments. IEEE Access.

[36] Kumari P., Singh A. (2019). Approximation and Updation of Betweenness Centrality in Dynamic Complex Networks. Computational Intelligence: Theories, Applications and Future Directions-Volume I.

[37] Hesse M., Dann D., Braesemann F., Teubner T. Understanding the Platform Economy: Signals, Trust, and Social Interaction. Proceedings of the Proceedings of the 53rd Hawaii International Conference on System Sciences.

